# Decreased miR-200b-3p in cancer cells leads to angiogenesis in HCC by enhancing endothelial ERG expression

**DOI:** 10.1038/s41598-020-67425-4

**Published:** 2020-06-26

**Authors:** Aye Moh-Moh-Aung, Masayoshi Fujisawa, Sachio Ito, Hiroshi Katayama, Toshiaki Ohara, Yoko Ota, Teizo Yoshimura, Akihiro Matsukawa

**Affiliations:** 1 Department of Pathology and Experimental Medicine, Graduate School of Medicine, Dentistry and Pharmaceutical Sciences, Okayama University, 2-5-1 Shikata, Kita-ku, Okayama, 700-8558 Japan; 2 Department of Molecular Oncology, Graduate School of Medicine, Dentistry and Pharmaceutical Sciences, Okayama University, 2-5-1 Shikata, Kita-ku, Okayama, 700-8558 Japan

**Keywords:** Tumour angiogenesis, Molecular medicine

## Abstract

Transcription factor ERG (erythroblast transformation-specific (ETS)-related gene) is essential in endothelial differentiation and angiogenesis, in which microRNA (miR)-200b-3p targeting site is expected by miRNA target prediction database. miR-200b is known decreased in hepatocellular carcinoma (HCC), however, the functional relation between ERG and miR-200b-3p, originating from pre-miR-200b, in HCC angiogenesis remains unclear. We investigated whether hepatocyte-derived miR-200b-3p governs angiogenesis in HCC by targeting endothelial ERG. Levels of miR-200b-3p in HCC tissues were significantly lower than those in adjacent non-HCC tissues. Poorly differentiated HCC cell line expressed lower level of miR-200b-3p compared to well-differentiated HCC cell lines. The numbers of ERG-positive endothelial cells were higher in HCC tissues than in adjacent non-HCC tissues. There was a negative correlation between the number of ERG-positive cells and miR-200b-3p expression in HCC tissues. Culture supernatants of HCC cell lines with miR-200b-3p-overexpression reduced cell migration, proliferation and tube forming capacity in endothelial cells relative to the control, while those with miR-200b-3p-inhibition augmented the responses. Exosomes isolated from HCC culture supernatants with miR-200b-3p overexpression suppressed endothelial ERG expression. These results suggest that exosomal miR-200b-3p from hepatocytes suppresses endothelial ERG expression, and decreased miR-200b-3p in cancer cells promotes angiogenesis in HCC tissues by enhancing endothelial ERG expression.

## Introduction

Globally, hepatocellular carcinoma (HCC) is one of the main causes of cancer-related death^1^. Similar to other cancers, angiogenesis is essential for cancer growth and metastasis in HCC. An angiogenic switch is always activated within tumors, which results in vascular hyperplasia^2^. Activation of angiogenic switches is regulated by various factors elicited by tumor cells or tumor microenvironments^3^. Recently, there is an increasing interest in understanding the tumor angiogenesis process as regulating angiogenesis is a potential target for cancer therapy.

In recent decades, there is a renewed interest in evaluating the roles of microRNAs (miRNAs) in cancer biology. miRNAs are small (18–22 nucleotides) non-coding RNA molecules, which modulate various cellular activities, such as proliferation, differentiation, apoptosis, and angiogenesis^4,5^. miRNAs regulate angiogenesis by targeting angiogenic factors and protein kinases^6^. Despite aberrant miRNAs expressions in HCC^7^, it is worthy to note that miR-200b is down-regulated in HCC tissues as compared to adjacent non-cancer tissues^8^. Recent studies demonstrated that miR-200b suppresses angiogenesis. Downregulation of endothelial miR-200b promotes the cutaneous wound angiogenesis^9^. miR-200b inhibits angiogenesis through direct and indirect mechanisms by targeting interleukin-8 and CXCL1 by tumor endothelial and cancer cells^10^. miR-200b silences several angiogenic growth factors and their receptors by directly targeting their mRNA transcripts^11^.

A detailed investigation on the role of miR-200b in angiogenesis is important to elucidate the pathological mechanism underlying HCC. Each miRNA regulates hundreds of genes, which can be predicted by miRNA target prediction database. Recently, miRNAs are known to target ERG (erythroblast transformation-specific (ETS)-related gene) in prostate cancer and colorectal cancer^12,13^. ERG plays an essential role in endothelial homeostasis, differentiation, and angiogenesis^14,15^, in which miR-200b-3p targeting site is expected by miRNA target prediction database. We hypothesized that down-regulated miR-200b-3p in HCC may cause enhanced endothelial ERG expression, leading to increased angiogenesis in the cancer microenvironment.

In this study, we, for the first time, demonstrate that ERG is a target of miR200b-3p and hepatocyte-derived miR-200b-3p reduces the endothelial cell migration, proliferation and tube forming capacity in endothelial cells. Additionally, we demonstrate that HCC tissues exhibit reduced miR-200b-3p expression, which causes augmented endothelial ERG expression, promoting angiogenesis in the cancer microenvironment. Furthermore, miR-200b-3p appears to be transferred by exosomes released from hepatocytes. Thus, miR-200b-3p can be a novel therapeutic target for the regulation of cancer angiogenesis.

## Results

### Expression of miR-200b-3p in HCC tissues and cell lines

**Figure 1:**
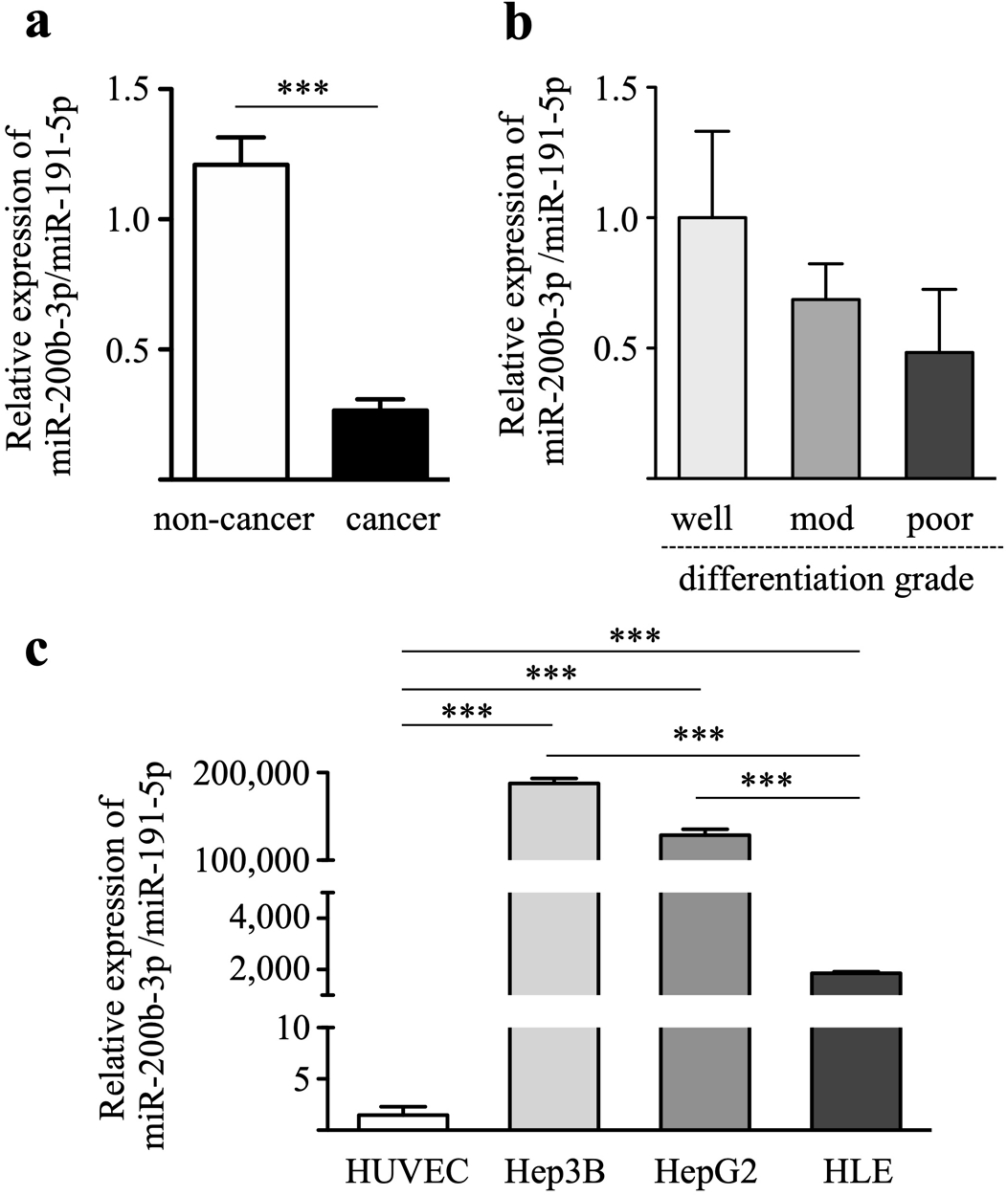
Expression level of miR-200b-3p in HCC tissues and cell lines. (**a**) The expression levels of miR-200b-3p in 40 pairs of HCC and adjacent non-cancer tissues were evaluated by qRT-PCR. ***p < 0.0001*.* (**b**) The expression levels of miR-200b-3p in the well (10 cases), moderately (26 cases), and poorly differentiated (4 cases) HCC groups were measured. (**c**) miR-200b-3p expression levels in HCC cell lines (Hep3B, HepG2, and HLE cells) and human umbilical vein endothelial cells (HUVECs) were quantitated. ***p < 0.0001.

The expression levels of miR-200b-3p were analyzed in forty pairs of clinical HCC and adjacent non-cancer tissues by qRT-PCR. The cases for the enrolled 40 patients with HCC are shown in Table 1. As shown in Fig. 1a, the miR-200b-3p expression levels in the HCC tissues were significantly lower than those in adjacent non-cancer tissues. The miR-200b-3p expression levels tended to be lower with decreasing grade of cancer differentiation although it was not statistically significant (Fig. 1b). Further detailed clinical data from enrolled patients with HCC were shown in Table 2. Among HCC with trabecular pattern, miR-200b-3p expression levels in moderately plus poorly differentiated HCC tended to be lower than those in well differentiated HCC (Table 2). Next, we examined the expression levels of miR-200b-3p in three HCC cell lines. The expression levels of miR-200b-3p in poorly differentiated cell line (HLE cell) were significantly lower than those in two well-differentiated HCC cell lines (Hep3B and HepG2 cells) (Fig. 1c). In contrast, the expression levels of miR-200b-3p in HUVECs was extremely low when compared to those in three HCC cell lines (Fig. 1c). These data indicate that non-cancer tissues, likely non-cancer hepatocytes, exhibit high levels of miR-200b-3p expression, whereas HCC tissues, likely cancer cells, exhibit decreased expression depending on the degree of cancer differentiation.

**Table 1:** Cases for the enrolled HCC patients.

Case	Age	Sex	Tumor stage	Growth pattern	Tumor grade	Cirrhosis
1	65	M	1b	Mixed	Moderate	−
2	71	M	1a	Trabecular	Well	−
3	75	F	1b	Pseudoglandular	Moderate	+
4	68	F	1b	Mixed	Poor	−
5	73	M	1b	Trabecular	Well	−
6	69	F	1b	Trabecular	Moderate	+
7	56	M	1a	Trabecular	Moderate	−
8	79	M	1b	Mixed	Moderate	+
9	61	M	2	Trabecular	Poor	−
10	74	M	1a	Solid	Moderate	+
11	79	M	2	Mixed	Moderate	−
12	55	M	1a	Trabecular	Well	+
13	82	F	2	Trabecular	Moderate	−
14	52	M	1a	Trabecular	Moderate	+
15	71	M	1b	Mixed	Moderate	−
16	66	M	2	Mixed	Well	+
17	62	M	1a	Trabecular	Moderate	+
18	62	F	2	Trabecular	Moderate	−
19	64	F	2	Trabecular	Well	+
20	68	M	2	Mixed	Moderate	−
21	59	M	1a	Pseudoglandular	Moderate	−
22	73	M	2	Trabecular	Moderate	−
23	61	F	1a	Mixed	Well	+
24	63	M	1a	Pseudoglandular	Well	−
25	73	F	1b	Mixed	Moderate	−
26	70	M	1b	Mixed	Moderate	−
27	70	M	1a	Trabecular	Moderate	−
28	69	M	1a	Trabecular	Moderate	+
29	60	F	1a	Trabecular	Well	+
30	62	M	1b	Trabecular	Moderate	−
31	57	M	1b	Mixed	Moderate	−
32	74	F	1a	Trabecular	Well	−
33	61	M	1b	Mixed	Moderate	−
34	64	F	1a	Trabecular	Well	+
35	57	F	1b	Solid	Poor	−
36	55	M	1b	Trabecular	Poor	+
37	69	M	1b	Mixed	Moderate	−
38	50	M	2	Trabecular	Moderate	+
39	77	F	2	Mixed	Moderate	−
40	69	F	1a	Trabecular	Moderate	+

Tumour stage, tumour grade and growth pattern are classified as according to WHO (2019) classification.

**Table 2:** The relationship between miR-200b-3p expression levels in cancer tissues and clinicopathological factors of patients with HCC.

Characteristics	Number of patients	Percentage of patients (%)	miR-200b-3p/miR-191-5p (mean ± SD)	*p* value
**Age (years)**				0.344^a^
> 70	26	65	0.293 ± 0.323	
≤ 70	14	35	0.218 ± 0.171	
**Gender**				0.409^b^
Male	26	65	0.293 ± 0.235	
Female	14	35	0.317 ± 0.352	
**Alcohol history**				0.415^b^
Yes	19	47.5	0.228 ± 0.227	
No	21	52.5	0.301 ± 0.321	
**Diabetes mellitus**				0.185^b^
Yes	13	32.5	0.182 ± 0.209	
No	27	67.5	0.308 ± 0.303	
**Performance status**				0.219^c^
0	24	60	0.255 ± 0.247	
1	11	27.5	0.203 ± 0.257	
2	5	12.5	0.463 ± 0.431	
**Child Pugh score**				0.124^c^
1	31	77.5	0.221 ± 0.231	
2	3	7.5	0.511 ± 0.354	
3	6	15	0.382 ± 0.417	
**Liver transferase (IU/L)**				0.354^b^
Low (AST ≤ 30 or ALT ≤ 25)	17	42.5	0.223 ± 0.162	
High (AST > 30 or ALT > 25)	23	57.5	0.299 ± 0.342	
**AFP (ng/mL)**				0.176^b^
≤ 10	22	55	0.212 ± 0.223	
> 10	18	45	0.333 ± 0.330	
**Platelets (× 10**^**4**^**/µL)**				0.346^b^
≤ 16	23	57.5	0.303 ± 0.309	
> 16	17	42.5	0.218 ± 0.234	
**Prothrombin time (s)**				0.302^b^
≤ 13	32	80	0.244 ± 0.249	
> 13	8	20	0.359 ± 0.385	
**Viral infection**				0.245^a^
Yes	29	72.5	0.289 ± 0.319	
No	11	27.5	0.208 ± 0.117	
**Diameter (cm)**				0.899^b^
≤ 2	17	42.5	0.260 ± 0.312	
> 2	23	57.5	0.272 ± 0.260	
**Multiplicity**				0.082^b^
Yes	5	12.5	0.470 ± 0.311	
No	35	87.5	0.238 ± 0.267	
**Differentiation**				0.447^c^
Well	10	25	0.358 ± 0.375	
Moderate	26	65	0.246 ± 0.250	
Poor	4	10	0.173 ± 0.174	
**Pattern**				0.401^c^
Trabecular	21	52.5	0.314 ± 0.312	
Solid	2	5	0.087 ± 0.097	
Pseudoglandular	3	7.5	0.064 ± 0.053	
Mixed	14	35	0.264 ± 0.256	
**Differentiation in trabecular**				0.081^b^
Well	7	33.3	0.482 ± 0.387	
Moderate + poor	14	66.7	0.230 ± 0.240	
**Grade in mixed**				0.352^b^
Well	2	14.3	0.101 ± 0.043	
Moderate + poor	12	85.7	0.291 ± 0.268	
**Microvascular invasion**				0.512^a^
Yes	6	15	0.437 ± 0.437	
No	34	85	0.247 ± 0.247	
**Cirrhosis**				0.381^b^
Yes	16	40	0.315 ± 0.340	
No	24	60	0.235 ± 0.233	
**TNM stage**				0.511^b^
I	29	72.5	0.248 ± 0.276	
II	11	27.5	0.315 ± 0.295	
**BCLC stage**				0.540^d^
0	4	10	0.204 ± 0.095	
A	16	40	0.257 ± 0.270	
B	1	2.5	0.682 ± 0.000	
C	13	32.5	0.212 ± 0.250	
D	6	15	0.382 ± 0.417	

*AFP*, *α-fetoprotein*, *BCLC* Barcelona Clinic Liver Cancer.

Statistical analyses: ^a^unpaired *t* test with Welch's correction, ^b^unpaired *t* test, ^c^one-way analysis of variance, ^d^Kruskal–Wallis test.

### Expression of ERG in HCC tissues

**Figure 2:**
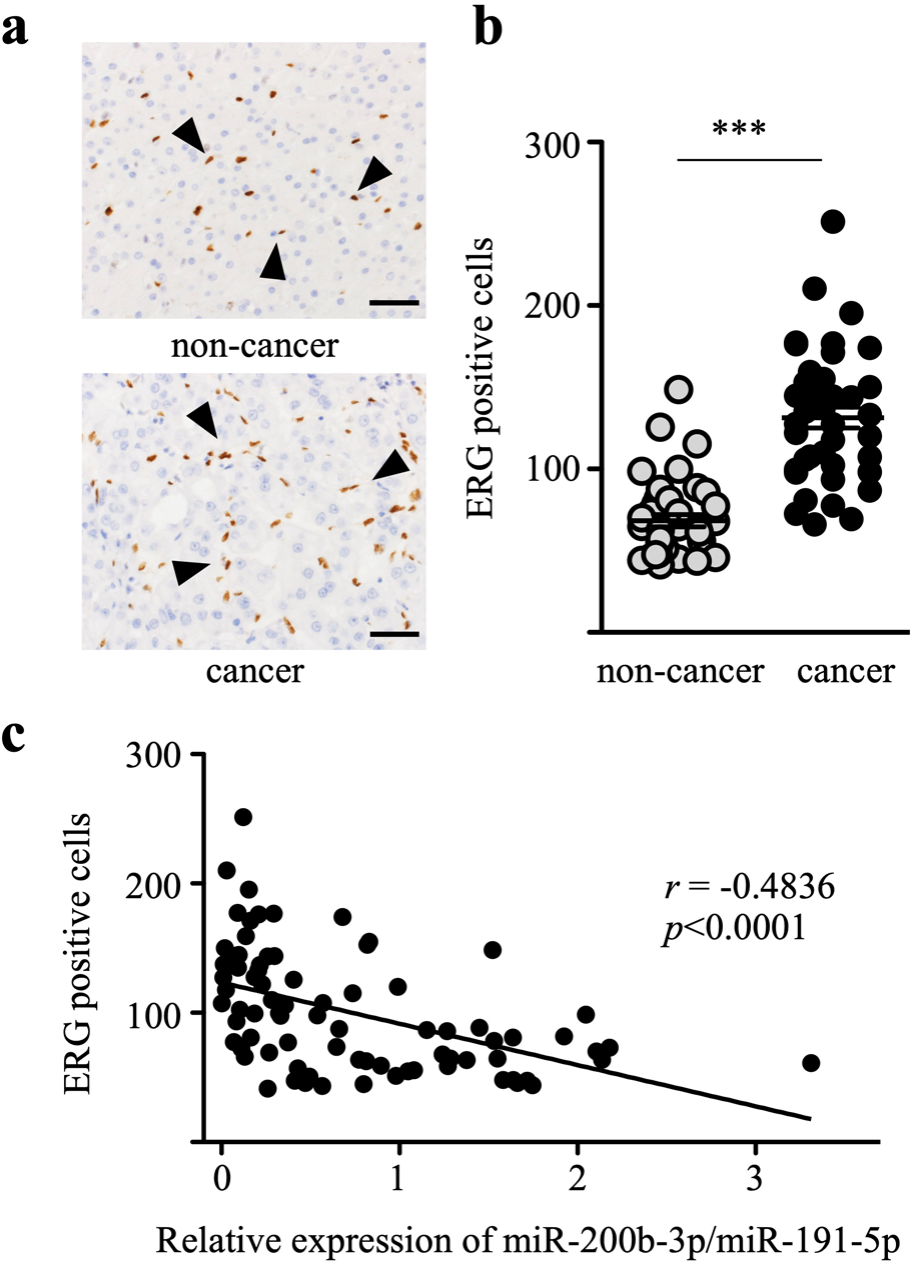
ERG-positive endothelial cells in HCC tissues. (**a**) Representative images of ERG-positive cells in a patient with moderately differentiated HCC. Arrowheads indicate positive staining of ERG in the nuclei of endothelial cells (scale bar, 50 μm). (**b**) The numbers of ERG-positive cells in the 40 pairs of non-cancer and cancer tissues from patients with HCC. ***p < 0.0001. (**c**) The number of ERG positive endothelial cells showed a negative correlation with the expression of miR-200b-3p in the HCC tissues (r = − 0.4836, *p* < 0.0001).

Expression of ERG in HCC tissues was next analyzed. As shown in Fig. 2a, nuclei of endothelial cells in tissue samples were stained positive for ERG. The numbers of ERG-positive endothelial cells in cancer tissue were more than those in adjacent non-cancer tissue (Fig. 2b). As shown in Fig. 2c, the statistical analysis indicated a negative correlation between the number of ERG-positive endothelial cells and the expression of miR-200b-3p in the HCC tissues (*r* = − 0.4836, *p* < 0.0001), suggesting that decreased miR-200b-3p expression in cancer tissue causes vascular hyperplasia in HCC tissues.

### miR-200b-3p negatively regulates endothelial ERG expression

**Figure 3:**
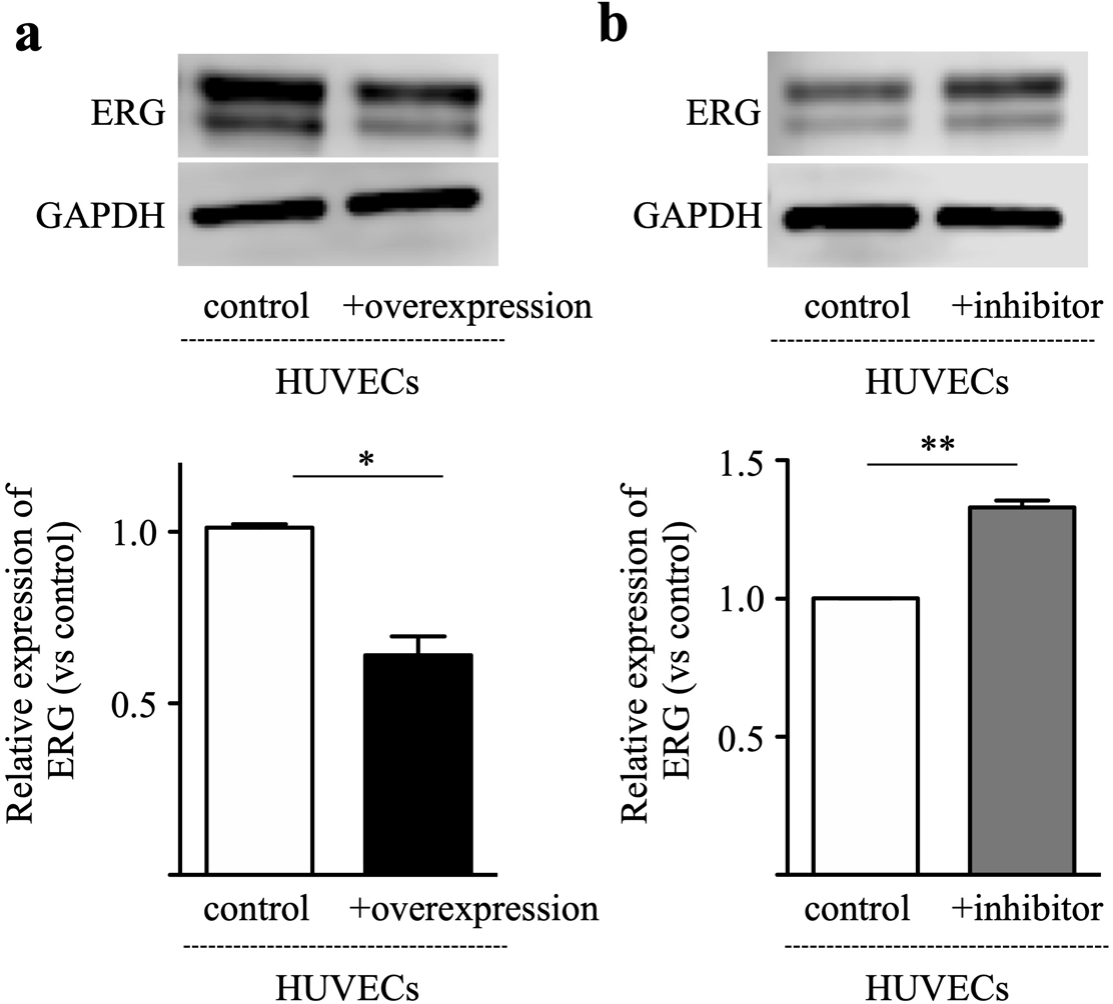
miR-200b-3p negatively regulates ERG expression in HUVECs. ERG protein expression in the HUVECs was analyzed by Western blotting. (**a**) miR-200b-3p was overexpressed in the HUVECs using an overexpression plasmid. miRNA control plasmid was used as a control. (**b**) miR-200b-3p was inhibited by miR-200b-3p inhibitor. miRNA inhibitor negative control was used as a control. Upper panel, representative immunoblot data from three independent analyses of HUVEC lysates. Lower panel, quantitative data are shown. *p < 0.01, **p < 0.001.

ERG is a transcription factor, and plays a crucial rule in vascular development, angiogenesis and vascular stability^14–16^. We assumed that miR-200b-3p may affect endothelial ERG expression. To investigate this assumption, miR-200b-3p was overexpressed in HUVECs, after which the cells were cultured with complete medium containing VEGF, and the ERG expression was examined by western blotting. As shown in Fig. 3a, levels of ERG in HUVECs overexpressing miR-200b-3p were significantly lower than those in controls. Although HUVECs exhibited much lower miR-200b-3p expression levels compared to HCC cell lines (Fig. 1c), inhibition of miR-200b-3p in HUVECs increased the ERG expression when compared to controls (Fig. 3b). These data show that miR-200b-3p partly downregulates endothelial ERG expression.

### ERG is a direct target of miR-200b-3p

**Figure 4:**
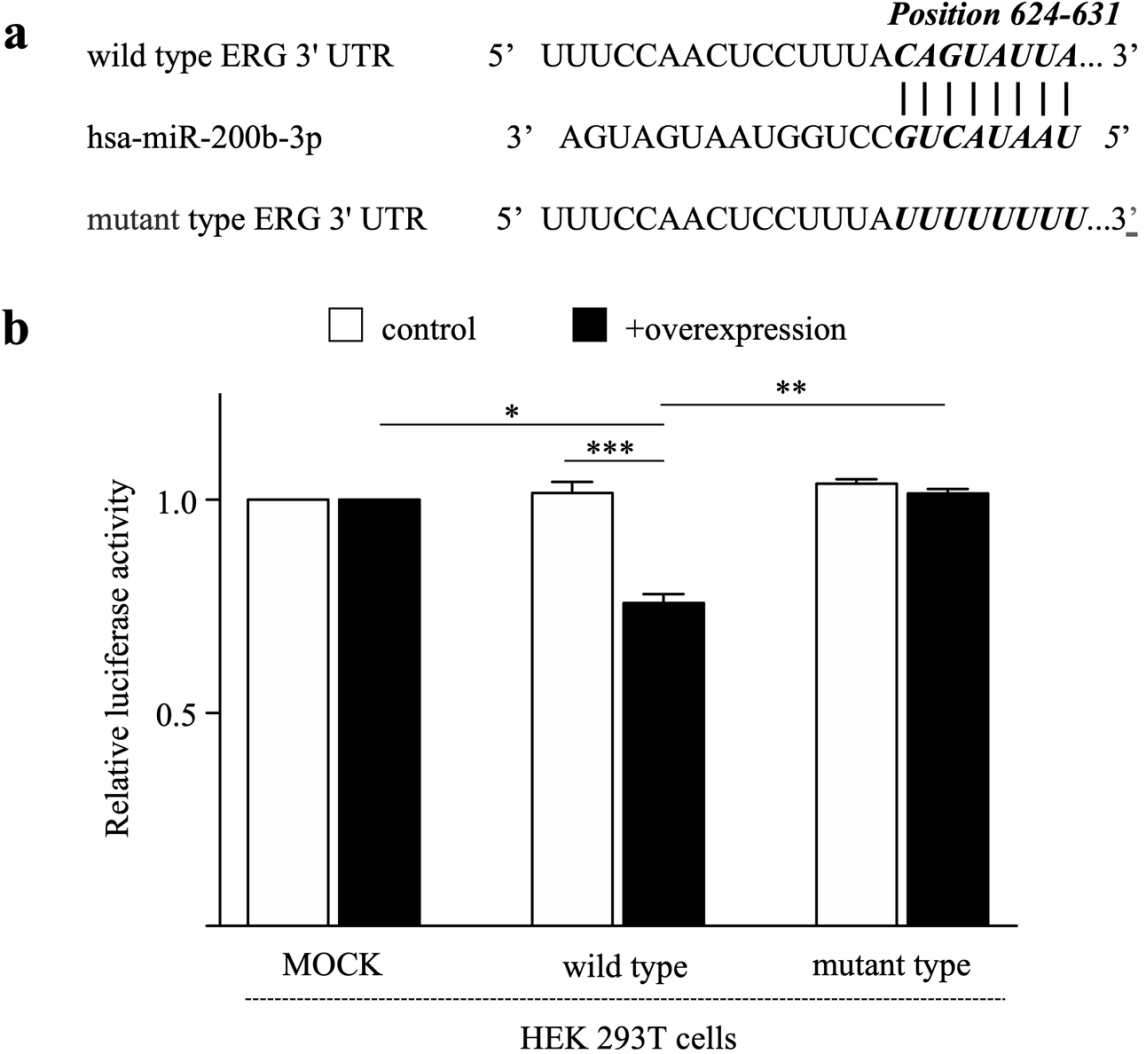
ERG is a direct target of miR-200b-3p. (**a**) The complementary sequences of miR-200b-3p in the ERG mRNA 3′-UTR. (**b**) Luciferase activity after transfection with constructs containing the ERG mRNA 3′-UTR (wild-type or mutant type) with or without miR-200b-3p expression plasmid was evaluated using dual-luciferase reporter system. *p < 0.01, **p < 0.001, ***p < 0.0001.

Analyzing the microRNA target prediction database and tools, such as TargetScanHuman (https://www.targetscan.org/vert_72/), microRNA.org (https://www.microrna.org/microrna/home.do) and miRDB (https://mirdb.org) revealed that the seed region of miR-200b-3p and 3′-UTR of ERG mRNA (position 624–631) are complementary (Fig. 4a). We generated an oligonucleotide of 23 base pairs containing miR-200b-3p binding sequence of ERG 3′-UTR and cloned into pmirGLO Dual-Luciferase miRNA Target Expression Vector and analyzed if ERG is a direct target of miR-200b-3p by using the dual-luciferase reporter assay. A similar construct lacking the target sequence of 3′-UTR of ERG was used as mutant construct. As shown in Fig. 4b, co-expression of precursor miRNA-200b-3p and wild type ERG 3′-UTR construct in HEK239T cells significantly reduced luciferase activity. There was no significant difference in luciferase activities when the ERG 3′-UTR sequence was mutated (*p* = 0.1686) (Fig. 4b). These data indicate that miR-200b-3p can directly target the 3′-UTR of the ERG gene and suppress the ERG expression.

### Hepatic miR-200b-3p suppresses endothelial ERG expression

**Figure 5:**
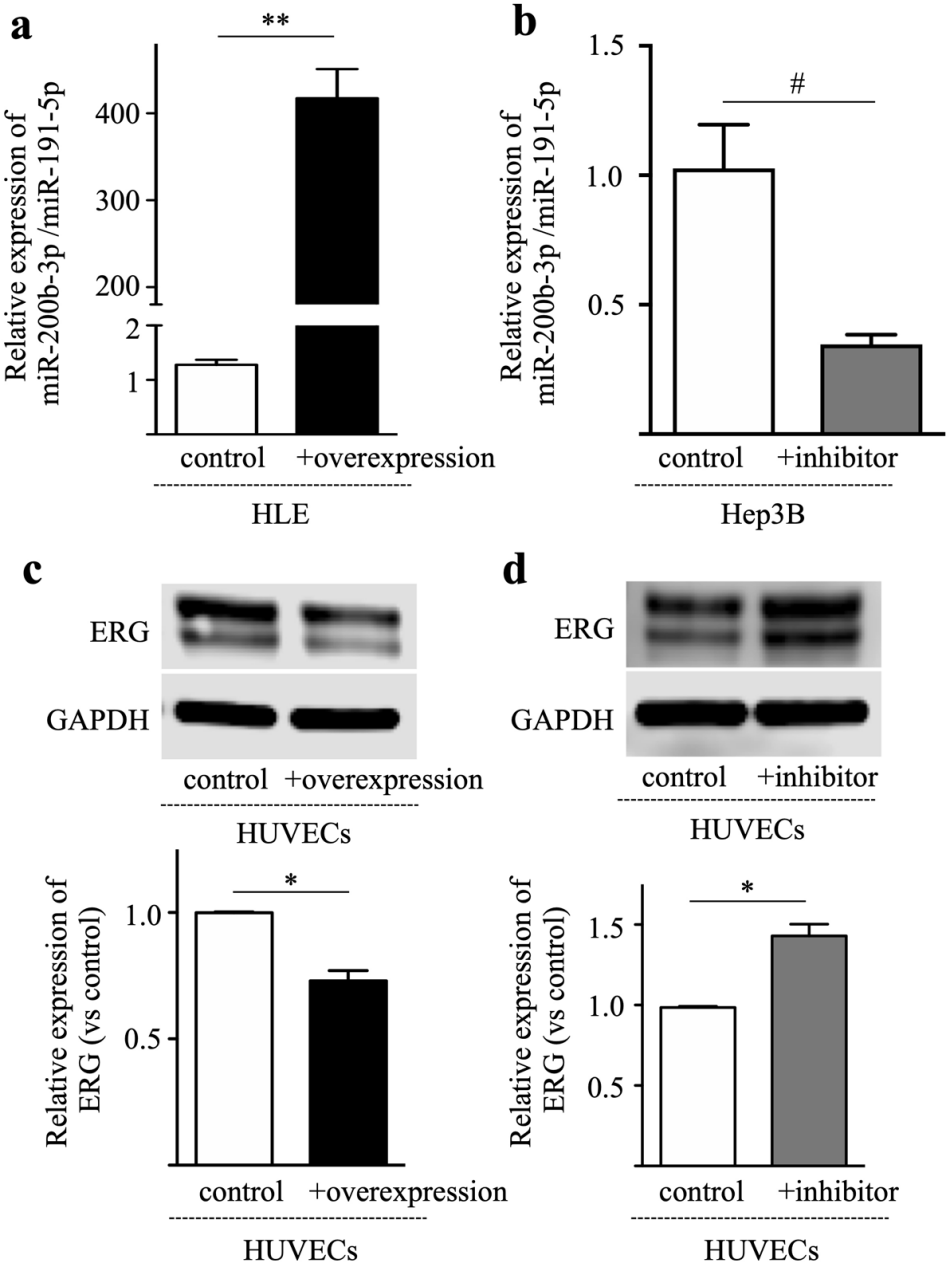
Hepatic miR-200b-3p suppresses ERG expression in HUVECs. (**a**) miR-200b-3p was overexpressed in HLE cells using an overexpression plasmid and relative expression of miR-200b-3p was measured. HLE cells transfected with control plasmid were used as a control. (**b**) miR-200b-3p was inhibited using miR-200b-3p inhibitor in the Hep3B cells and relative expression of miR-200b-3p was measured. Hep3B cells transfected with miRNA inhibitor negative control were used as a control. (**c**) HUVECs were cultured in the presence of culture supernatants of miR-200b-3p-overexpressing HLE cells or control medium transfected with miRNA control plasmid. Upper panel, representative immunoblot data from three independent analyses of HUVEC lysates. Lower panel, relative expression of ERG. (**d**) HUVECs were cultured in the presence of culture supernatants of miR-200b-3p-inhibited Hep3B cells or control medium transfected with miRNA inhibitor negative control. Upper panel, representative immunoblot data from three independent analyses of HUVEC lysates. Lower panel, relative expression of ERG. ^#^*p* < 0.05, *p < 0.01, **p < 0.001.

To examine the relation between hepatic miR-200b-3p and endothelial ERG, HUVECs were cultured with two types of HCC culture supernatants. For gain-of-function analysis, we overexpressed miR-200b-3p in HLE cells (miR-200b-3p expression; low, Fig. 1c) and harvested culture supernatants at 48 h (Fig. 5a). For loss-of-function experiments, we treated Hep3B cells (miR-200b-3p expression; high, Fig. 1c) with a specific inhibitor or inhibitor control and harvested culture supernatants (Fig. 5b). The culture supernatants were mixed with complete medium at a ratio of 1:1, and the HUVECs were cultured. As shown in Fig. 5c, the protein expression levels of ERG in HUVECs cultured with miR-200b-3p overexpression supernatants were lower than those with the control. By contrast, the protein expression levels of ERG in HUVECs treated with culture supernatants of miR-200b-3p-inhibition were higher than those with control (Fig. 5d). This suggests that hepatocyte-derived miR-200b-3p downregulates ERG expression in HUVECs.

### Hepatic miR-200b-3p inhibits angiogenesis

**Figure 6:**
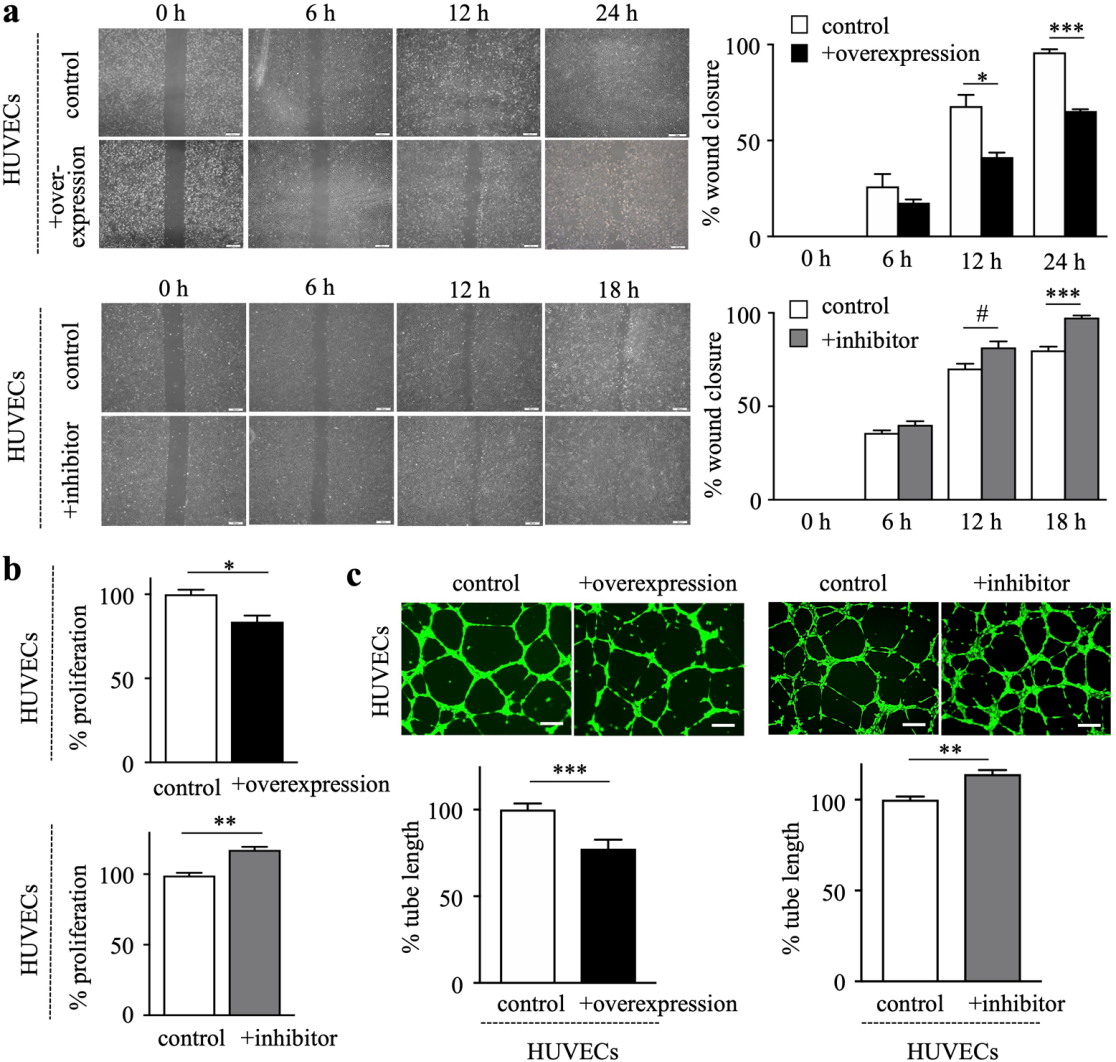
Hepatic miR-200b-3p inhibits angiogenic capability of HUVECs*.* (**a**) Wounds were generated by scratching with a sterile 200-µL tip. The HUVECs were cultured for 24 h with culture supernatants of miR-200b-3p-overexpression (upper left), miR-200b-3p-inhibitition (lower left), transfected with miRNA control plasmid or miRNA inhibitor negative control, respectively. Representative images of wound healing assay were shown at indicated time after scratch. Right, wound distance at different time points was measured and presented as the percentage of wound closure at time 0. (**b**) The HUVECs were cultured with culture supernatants of miR-200b-3p-overexpression (upper), miR-200b-3p-inhibition (lower) for 24 h and cell proliferation was assessed by XTT assay. (c) The HUVECs were seeded on Matrigel-coated plates and cultured with culture supernatants of miR-200b-3p-overexpression (left), miR-200b-3p-inhibitition (right). Shown are representative images of tube formation after 6 h (scale bar, 200 μm). Experiments were performed in triplicates. ^#^*p* < 0.05, *p < 0.01, **p < 0.001, ***p < 0.0001.

Angiogenesis requires not only endothelial cell proliferation but also their migration and formation of tubes. Scratch wound healing assay was performed using HUVECs to examine the role of miR-200b-3p in endothelial cell migration. Culture supernatants with miR-200b-3p overexpression inhibited the closure of HUVECs when compared to control (Fig. 6a, upper). In contrast, culture supernatants with miR-200b-3p-inhibition accelerated the wound closure (Fig. 6a, lower). miR-200b-3p-overexpressing culture supernatants decreased the proliferation of HUVECs (Fig. 6b, upper), which was increased by culture supernatants with miR-200b-3p-inhibition (Fig. 6b, lower). Next, tube formation assay was performed to evaluate the ability of HUVECs to form capillary-like structures. HUVECs cultured with miR-200b-3p-overexpressing supernatants exhibited reduced capacity to form capillary-like structures (Fig. 6c, left), whereas miR-200b-3p-inhibited supernatants increased the capacity when compared to control (Fig. 6c, right). These data suggest that hepatic miR-200b-3p downregulates angiogenic capability of HUVECs.

### miR-200b-3p is transferred by exosomes

**Figure 7:**
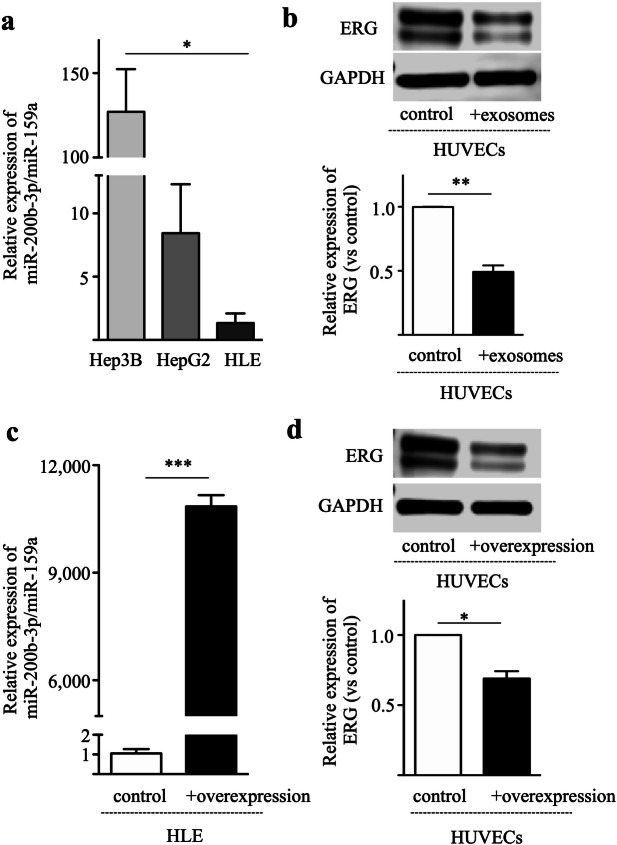
miR-200b-3p is transferred via exosomes. (**a**) Exosomes were isolated from the HCC cell lines and the expression levels of miR-200b-3p in the exosomes were measured. (**b**) HUVECs were cultured with or without exosomes isolated from HLE cell culture supernatants, and the ERG expression levels in HUVECs were investigated by Western blotting. Upper panel, representative immunoblot data from three independent analyses of HUVEC lysates. Lower panel, relative expression of ERG. (**c**) HLE cells were transfected with miR-200b-3p overexpression plasmid or control plasmid and the exosomes were isolated from the culture supernatants. miR-200b-3p expression levels in the exosomes were quantified by qRT-PCR. (**d**) HUVECs were cultured with exosomes isolated from miR-200b-3p-overexpressing HLE cell culture supernatants, and the ERG expression levels in HUVECs were investigated by Western blotting. Culture supernatants from HLE cells transfected with control plasmid were used as a control. Upper panel, representative immunoblot data from three independent analyses of HUVEC lysates. Lower panel, relative expression of ERG. *p < 0.01, **p < 0.001, ***p < 0.0001.

Exosomes are a heterogeneous population of vesicles that originate from cells. Exosomes play a key role in the intercellular communication between cancer cells and stroma cells^17,18^. Hence, we examined if exosomes released from cancer cells could transfer miR-200b-3p to endothelial cells. Exosomes were isolated from HCC cell lines and expression levels of miR-200b-3p in exosomes were measured. The expression levels of miR-200b-3p in the exosomes were high in Hep3B cells and low in HLE cells (Fig. 7a), which were proportional to those detected in cells (Fig. 1c). Next, HUVECs were cultured with exosomes isolated from HLE cell culture supernatants and expression levels of ERG in HUVECs were measured. As shown in Fig. 7b, addition of exosomes suppressed the expression of ERG in HUVECs as compared with control (no exosomes). To confirm the role of exosome-associated miR-200b-3p in inhibition of ERG expression in HUVECs, we overexpressed miR-200b-3p in HLE cells, and exosomes were isolated from miR-200b-3p-overexpressing HLE cell culture supernatants (Fig. 7c). Exosomes isolated from miR-200b-3p-overexpressing HLE cell culture medium significantly reduced ERG expression in HUVECs when compared to control (Fig. 7d). These data suggest that miR-200b-3p is transferred via exosomes from hepatocytes to endothelial cells, resulting in suppression of endothelial ERG expression.

## Discussion

Angiogenesis is necessary for cancer growth and metastasis and regulated by various molecules. In this study, we investigated the role of miR-200b-3p in regulating cancer angiogenesis in HCC. We used 40 paired human HCC tissues and adjacent non-cancer liver tissues and examined the expression of miR-200b-3p. We used Taqman advanced miRNA cDNA synthesis kit and a pair of advanced miRNA control to avoid amplification bias and reflect true expression level of mature miRNA. miR-200b is known decreased in hepatocellular carcinoma (HCC)^19,20^. In the present study, we focused on miR-200b-3p, which originates from pre-miR-200b. Our results demonstrated that clinical HCC specimens exhibited lower expression levels of miR-200b-3p than the surrounding non-cancer liver tissues. Interestingly, this lower expression of miR-200b-3p was associated with poor tumor differentiation, especially in HCC with trabecular pattern. Since miR-200b-3p is reported to inhibit angiogenesis^9,10,21,22^, we hypothesized that downregulation of miR-200b-3p in cancer cells may drive blood vessel hyperplasia in HCC.

miR-200b and other miR-200 family members (miR-200a/429) are reported to be ERG targeting miRNAs in prostate cancer^23^ and are involved in promoting cancer growth and invasion. ERG is an essential regulator of endothelial homeostasis and tumor angiogenesis^24^, but there is no study about ERG as miRNA-200b-3p target. In this study, we demonstrated that ERG is a miR-200b-3p target. The numbers of ERG positive endothelial cells in highly vascular cancer tissues were upregulated when compared to those in non-cancer tissues. Our in vitro experiments manipulating miR-200b-3p expression revealed that the decreased levels of miR-200b-3p in HCC tissues drive ERG expression in HUVECs. We know from the literature that angiogenesis in cancer tissues is regulated by various molecules. Among them, our data suggest that decreased levels of miR-200b-3p in cancer hepatocytes partly contribute to angiogenesis in HCC tissues.

Cancer cells are known to release large numbers of exosomes, which deliver molecules that have a pathogenic role in cancer pathology^25,26^. Exosomes are heterogeneous membrane-enclosed structures released by cells. Exosomes mediate both autocrine and paracrine signal transduction by transferring proteins, RNA, and miRNAs^27,28^. Recent evidence indicates that exosomes promote HCC cell proliferation, growth, invasion, and metastasis, as well as the development of drug resistance^29^. In this study, we demonstrated evidence that miR-200b-3p is released from hepatocytes inside exosomes and transferred to endothelial cells. Although we did not quantify levels of exosomes from non-tumor and tumor hepatocytes, the expression levels of miR-200b-3p in cancer area were quite lower than those in non-cancer area. It is likely that lower expression levels of exosomal miR-200b-3p is due to decreased expression of miR-200b-3p in HCC cells.

Our data indicate that hepatocyte-derived miR-200b-3p inhibits ERG expression in HUVECs. However, its effect was partial as assessed by overexpression or inhibition of miR-200b-3p. Although ERG is a target of miR-200b-3p in endothelial cells, a single transcript may be regulated by multiple miRNAs, as each miRNA is known to regulates hundreds of genes. For instance, miR-196a and miR-196b induced ERG downregulation in leukemia^30^. ERG is a target of miR-145 in colorectal cancer and prostate cancer^12,31^. Alternatively, angiogenic growth factors, including vascular endothelial growth factor (VEGF), platelet-derived growth factor (PDGF), and fibroblast growth factor (FGF), released from HCC cells^32^ may affect ERG expression in HUVECs. Thus, ERG expression in endothelial cells is regulated by multiple mechanisms and a single inhibition of miR-200b-3p has a limited effect. Regulation of ERG by miRNA-200b-3p in other cancers is a subject for future investigation.

In this study, we did not evaluate the mechanisms underlying the downregulation of miR-200b-3p in HCC cells. Recent studies have revealed an importance of epigenetic control in the expression of distinct miRNAs. The expression of the miR-200 family is reported to be regulated by DNA methylation and histone modifications^33^. The promoter methylation status of miR-200b is reported to determine tumor outcome in gastric cancer^34^. The hypo-methylated promoter enhances miR-200b expression in pancreas cancer^35^. Methylation-specific PCR (MSP) and bisulfite sequencing PCR (BSP) revealed that CpG sites in the promoter region of microRNA-200b were extensively methylated in HCC cells with concomitant downregulation of microRNA-200b expression^8^. Thus, epigenetic control appears to be important in the regulation of miR-200b-3p expression in HCC cells. Alternatively, cancer microenvironment including hypoxia may affect miR-200b-3p expression. Previous studies have reported various miRNAs that are differentially expressed in response to hypoxia, among which miR-200b was reported to be downregulated^36^. Further studies are necessary to understand the precise mechanisms underlying downregulation of miR-200b-3p in HCC cells.

**Figure 8:**
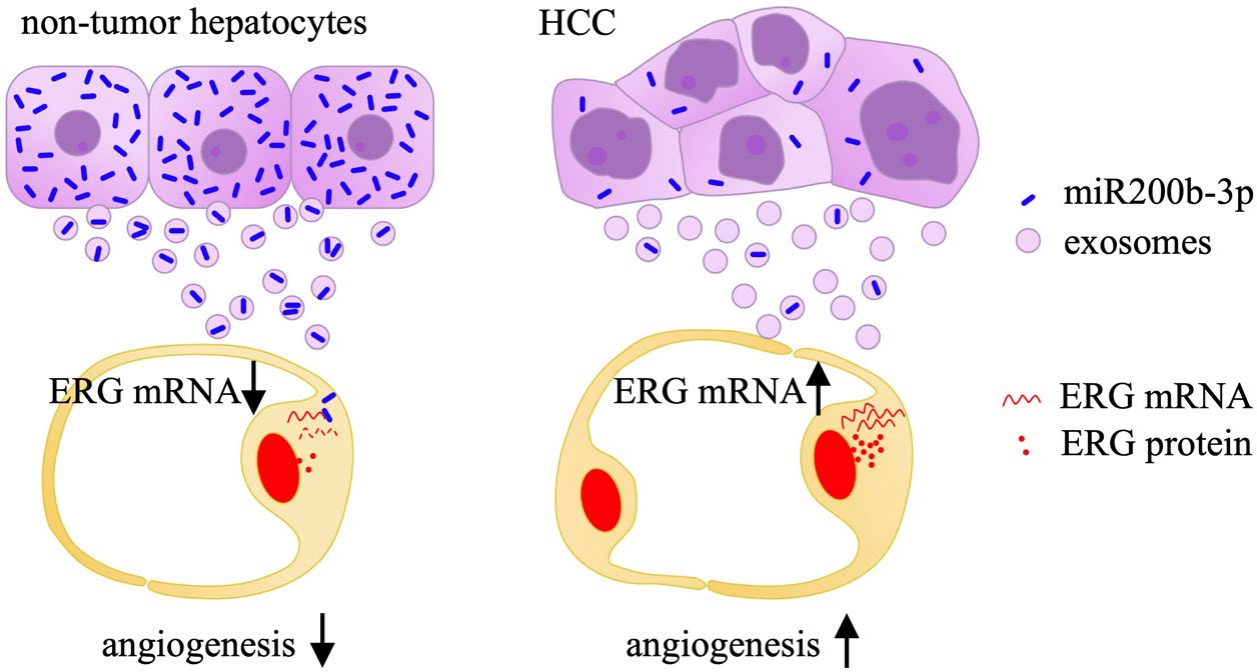
A schematic illustration indicating the role of miR-200b-3p in angiogenesis in HCC tissues. Left panel: In non-cancer tissues, hepatocytes generate and release miR-200b-3p via exosomes, which inhibits ERG protein expression in endothelial cells. Decreased expression of ERG inhibits the proliferation of endothelial cells. Right panel: In HCC tissues, miR-200b-3p expression by HCC cells is suppressed, and the ERG expression in endothelial cells is no longer inhibited. As a result, ERG expression is augmented, and angiogenesis is increased in HCC tissues.

The results of our study are illustrated in Fig. 8. In non-tumor tissues, exosomal miR-200b-3p released from hepatocytes downregulates vascular proliferation by inhibiting ERG expression in endothelial cells. By contrast, miR-200b-3p expression in HCC cells is downregulated, which drives vascular proliferation by enhancing ERG expression in endothelial cells. Hence, miR-200b-3p may be a novel molecular target for the treatment of HCC.

## Method

### Human tissue samples

In this study, we employed 40 pairs of tumor and adjacent non-tumor tissue derived from patients with HCC, who underwent surgical resection between January 2015 and December 2016 at Okayama University Hospital. The patients who underwent chemotherapy or radiotherapy before the resection were not included in this study. All the hematoxylin and eosin-stained tumor glass slides used for diagnosis were reviewed and the degree of differentiation of cancer was recorded. One representative tumor slide was selected for each case and the block was used for immunohistochemical studies. The protocol in this study was reviewed and approved by the *Ethics Committee* of *Okayama University *(*1703-007*). Although individual written consents were not obtained, we disclosed the study plan on our website, providing the patients or their families with the opportunity to opt out, and only the cases without their refusal were enrolled in the study. This consent procedure conformed to amended Ethical Guidelines for Clinical Studies provided by Ministry of Health, Labor and Welfare of Japan (May 31, 2015) and was approved by the institutional review boards. All methods were performed in accordance with the relevant guidelines and regulations.

### Cell culture

Hep3B cells (DS Pharma Biomedical, Osaka, Japan) were cultured in Eagle’s minimal essential medium (MEM) (Sigma-Aldrich, St. Louis, MO, USA) supplemented with MEM non-essential amino acid solution, 10% fetal bovine serum (FBS) (HyClone, UT, USA), 100 U/mL penicillin, and 100 μg/mL streptomycin (Sigma-Aldrich). HepG2 and HLE cells (JCRB cell bank, Osaka, Japan) were cultured in Dulbecco’s modified Eagle medium (Nacalai Tesque, Kyoto, Japan) supplemented with 10% FBS, 100 U/mL penicillin and 100 μg/mL streptomycin. Human umbilical vein endothelial cells (HUVECs) (Takara Bio Inc, Shiga, Japan) were cultured in EndoGRO™-VEGF Complete medium (Millipore, NJ, USA) in the absence of antibiotics. All cell lines were incubated at 37 °C and 5% CO_2_ in a humidified incubator.

### Transfection

Transfection was performed using Lipofectamine 3000 or Lipofectamine RNAiMax (Life Technologies, Carlsbad, CA, USA) in OPTI-MEM 1X reduced serum medium (Gibco, MD, USA) for 48 h. For gain-of-function analysis, 2.5 µg of precursor miR-200b-3p expression plasmid (HmiR0001-MR04, GeneCopoeia, MD, USA) or precursor miRNA scrambled control plasmid (CmiR0001-MR04, GeneCopoeia) was used for HUVECs and HLE cells (native miR-200b-3p expression; low) in 6-well plate. For loss-of-function experiments, 200 pmol mirVana hsa-miR-200b-3p inhibitor (4464084, Ambion, TX, USA) or 40 pmol mirVana miRNA inhibitor Negative Control #1 (4464076, Ambion) was introduced into the HUVECs and Hep3B cells (native miR-200b-3p expression; high) cultured in 6-well plate.

### Preparation of culture supernatants

miR-200b-3p-overexpressing HLE cells and miR-200b-3p-inhibited Hep3B cells were cultured for 48 h. Culture supernatants were harvested and centrifuged at 2,000*g* for 10 min at 4 °C, and the resultant clear culture supernatants were stored at − 80 °C until use.

### Quantitative real-time PCR (qRT-PCR) of miRNAs

Total RNA was extracted from human formalin-fixed paraffin-embedded (FFPE) tissues, cell lines, and exosomes using the RecoverAll™ Total Nucleic Acid Isolation Kit (Invitrogen, CA, USA), *mir*Vana™ miRNA Isolation Kit (Invitrogen), and Total Exosome RNA and Protein Isolation Kit (Invitrogen), respectively, following the manufacturer’s instructions. cDNA was synthesized from total RNA using TaqMan Advanced miRNA cDNA Synthesis kit (Applied Biosystems, Foster City, CA, USA). qRT-PCR analysis was performed on StepOnePlus real-time PCR system with TaqMan advanced miRNA assays (Applied Biosystems). To detect miR-200b-3p expression, hsa-miR-200b-3p (477963-mir) was used as the primer. According to the manufacturer’s instructions, a set of controls, miR-191-5p (477952-mir)^37^ and miR-26a-5p (477995-mir), was used to validate the expression levels of miR-200b-3p from FFPE tissues and cell lines. miRNAs including miR-191-5p and miR-26a-5p were statistically superior to the most commonly used reference RNAs used in miRNA qRT-PCR experiments, such as 5S rRNA, U6 snRNA, or total RNA^38^. The relative expression levels validated by miR-191-5p and miR-26a-5p were constantly similar (not shown). Therefore, we have shown the expression data relative to miR-191-5p. miR-200b-3p expression in the exosomes was normalized using 1 µL of 1 pM spike-in control, ath-miR-159a (478411-mir). All primers were purchased from Applied Biosystems. All experiments were performed in triplicates and the relative expression was calculated using the ∆∆CT method.

### Immunohistochemistry

Immunostaining was performed using the Histofine Simple Stain MAX-PO (Nichirei Biosciences Inc, Tokyo, Japan), following the manufacturer’s instructions. Briefly, the sections (4 µm slices) were deparaffinized, rehydrated, and subjected to heat-induced epitope retrieval in 0.5 M EDTA buffer (pH 8.0). Next, the sections were treated with 0.3% H_2_O_2_ in methanol and incubated with rabbit monoclonal anti-ERG antibody (1:1) (Histofine, Tokyo, Japan) for 90 min at room temperature. The sections were rinsed and incubated with peroxidase-conjugated secondary antibodies at room temperature for 45 min. Diaminobenzidine (DAKO, Carpinteria, CA, USA) was used as a chromogen. The images of four random consecutive areas (each area, 371 µm^3^) were captured under 20 × magnification of a light microscope. The number of ERG-positive nuclei in the endothelial cells was counted using the Image J software.

### Dual-luciferase reporter assay

HEK 293T cells (ATCC, VA, USA) were seeded in 12-well plates and cultured in DMEM supplemented with 10% FBS and antibiotics until the cells reached approximately 80% confluency. Synthetic oligonucleotides of 23 base pairs containing miR-200b-3p binding sequence were designated as wild type ERG 3′-UTR (Thermo Fisher Scientific, MA, USA), which was cloned into the pmirGLO Dual-Luciferase miRNA Target Expression Vector (Promega, WI, USA) at *Xba*I restriction enzyme site. Mutant type ERG 3′-UTR constructs were also generated by replacing the targeting site of seed region with thymine (T) (Thermo Fisher Scientific). HEK 293 T cells were transiently co-transfected with 50 ng of luciferase reporter construct (empty vector as control or ERG wild type or ERG mutant type) along with 1 µg of precursor miRNA-200b-3p expression plasmid or precursor miRNA scrambled control plasmid using Lipofectamine 3000, following the manufacturer’s instructions. Each sample was cultured in duplicates for 48 h. The cell lysates were harvested to determine the firefly and Renilla luciferase activity using a Dual-Luciferase Reporter Assay System (Promega) with Fluoroskan Ascent FL (Thermo Fisher Scientific). The luminescence value was normalized by calculating the ratio of firefly luminescence to Renilla luminescence. Three independent experiments were performed.

### Isolation of exosomes

Confluent cells were washed with phosphate buffer saline (PBS) twice and cultured in serum-free medium for 24 h. The culture media were collected and centrifuged at 2000*g* and 4 °C for 10 min. Supernatant was filtered through a 0.2-µm syringe filter. The exosomes were isolated using the Total Exosome Isolation kit (from cell culture media) (Thermo Fisher Scientific), following the manufacturer’s instructions. Briefly, 0.5 volumes of the Total Exosome Isolation reagent were mixed with the culture supernatant and incubated at 4 °C overnight. The samples were centrifuged at 10,000*g* and 4 °C for 1 h and the supernatant was discarded. The pellets were resuspended in D-PBS(–) for HUVEC culture or Exosome Resuspension Buffer (Thermo Fisher Scientific) for isolation of miR-200b-3p.

### Western blotting

The cells were lysed in RIPA buffer containing protease inhibitor. The total protein concentrations were measured using the BCA Protein Assay Kit (TaKaRa). Equal amounts of lysates were loaded per lane on 4–12% polyacrylamide gels (Thermo Fisher Scientific). The proteins were resolved by sodium dodecyl sulfate (SDS)-polyacrylamide gel electrophoresis. The resolved proteins were transferred onto nitrocellulose membranes (pore size, 0.45 µm). The membrane was incubated with anti-ERG antibody (1:10) or anti-GAPDH antibody (1:5,000) (Cell Signaling, MA, USA) overnight. Next, the membrane was incubated with the horseradish peroxidase-conjugated rabbit IgG antibody (1:1,000) (Cell Signaling, MA, USA). The proteins were visualized using the enhanced chemiluminescence detection reagents (ImmunoStar LD; FUJIFILM Wako, Osaka, Japan). The protein band intensity was quantified using Image Studio Lite software. All experiments were repeated in triplicates.

### Scratch wound healing assay

The HUVECs were seeded in a 6-well plate to obtain the confluent monolayer. The wounds were generated by scratching the monolayer with a sterile 200-µL tip. The wounded cell monolayers were cultured for 24 h. The wound distance at different time points was measured by Image J software and presented as the percentage of wound closure at time 0. The experiments were performed in triplicates.

### Proliferation assay

HUVECs were cultured in 96-well plate for 24 h. 50 µL of XTT labeling mixture (final XTT concentration 0.3 mg/mL, Cell proliferation Kit II, Sigma-Aldrich) was added into each well. After incubation at 37 °C for 1 h, the absorbance of each well was measured at 480–650 nm by using ELISA reader. Each sample was tested in duplicates, and the experiments were performed in triplicates.

### Tube formation assay

The HUVECs were starved for 6 h in Medium 200 Phenol Red Free medium (Gibco). The cells (4 × 10^4^/well) were seeded onto the 24-well plate precoated with Matrigel Basement Membrane Matrix (Corning, NY, USA) and incubated with culture supernatants containing 1% FBS for 6 h. Next, the cells were incubated with 2 μg/mL Calcein (Invitrogen) for 30 min. The tube formation was visualized and photographed using the BZ-X700 fluorescence microscope (Keyence Corp, Osaka, Japan). The total tube length was measured by cellSens standard under 10X magnification. Each treatment was performed in triplicates, and 4 visions were counted to obtain an average.

### Statistical analysis

The data were analyzed using the GraphPad Prism software (GraphPad Software, San Diego, CA, USA). Student *t* test was used to determine the statistical significance. Data are presented as mean ± standard error of mean (SEM). The relation between the miR-200b-3p expression and number of ERG positive cells in HCC tissues was assessed by using the Pearson’ s relation coefficient. Clinical data were analysed by unpaired t test, unpaired t test with Welch's correction, one-way analysis of variance, and Kruskal–Wallis test. The difference was considered statistically significant when the *p* value was less than 0.05 for all experiments.

## Supplementary information

Supplementary information.
