# Supplementary material for: Decreased miR-200b-3p in cancer cells leads to angiogenesis in HCC by enhancing endothelial ERG expression
Source: Sci Rep. 2020 Jun 26;10:10418. doi: 10.1038/s41598-020-67425-4 (PMC7320004; doi:10.1038/s41598-020-67425-4)

TITLE:

Decreased miR-200b-3p in cancer cells leads to angiogenesis in HCC by enhancing endothelial ERG expression

AUTHORS:

Aye Moh-Moh-Aung, Masayoshi Fujisawa, Sachio Ito, Hiroshi Katayama, Toshiaki Ohara, Yoko Ota, Teizo Yoshimura, Akihiro Matsukawa

3A, HUVECs

ERG

GAPDH

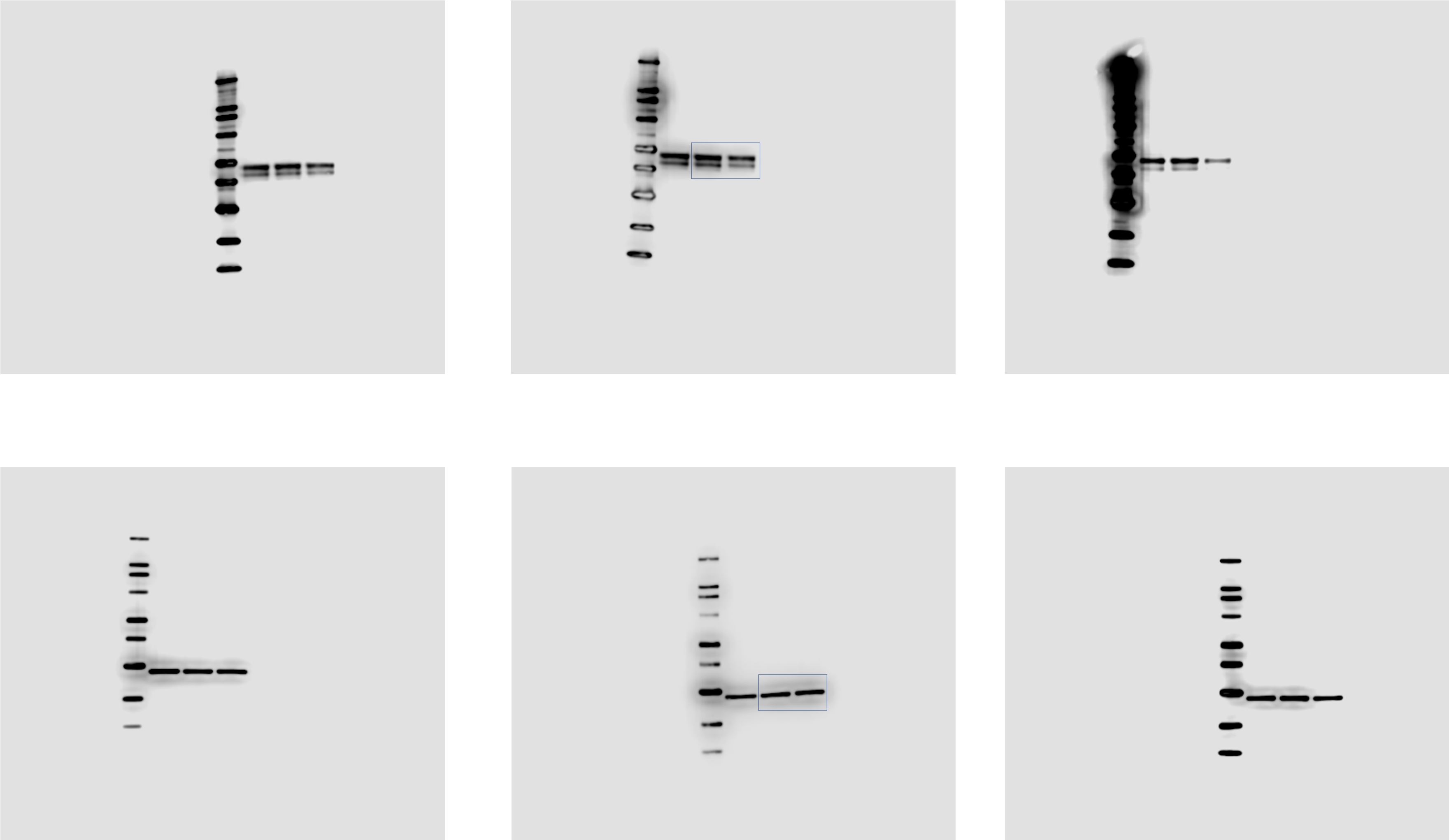

3B, HUVECs

ERG

GAPDH

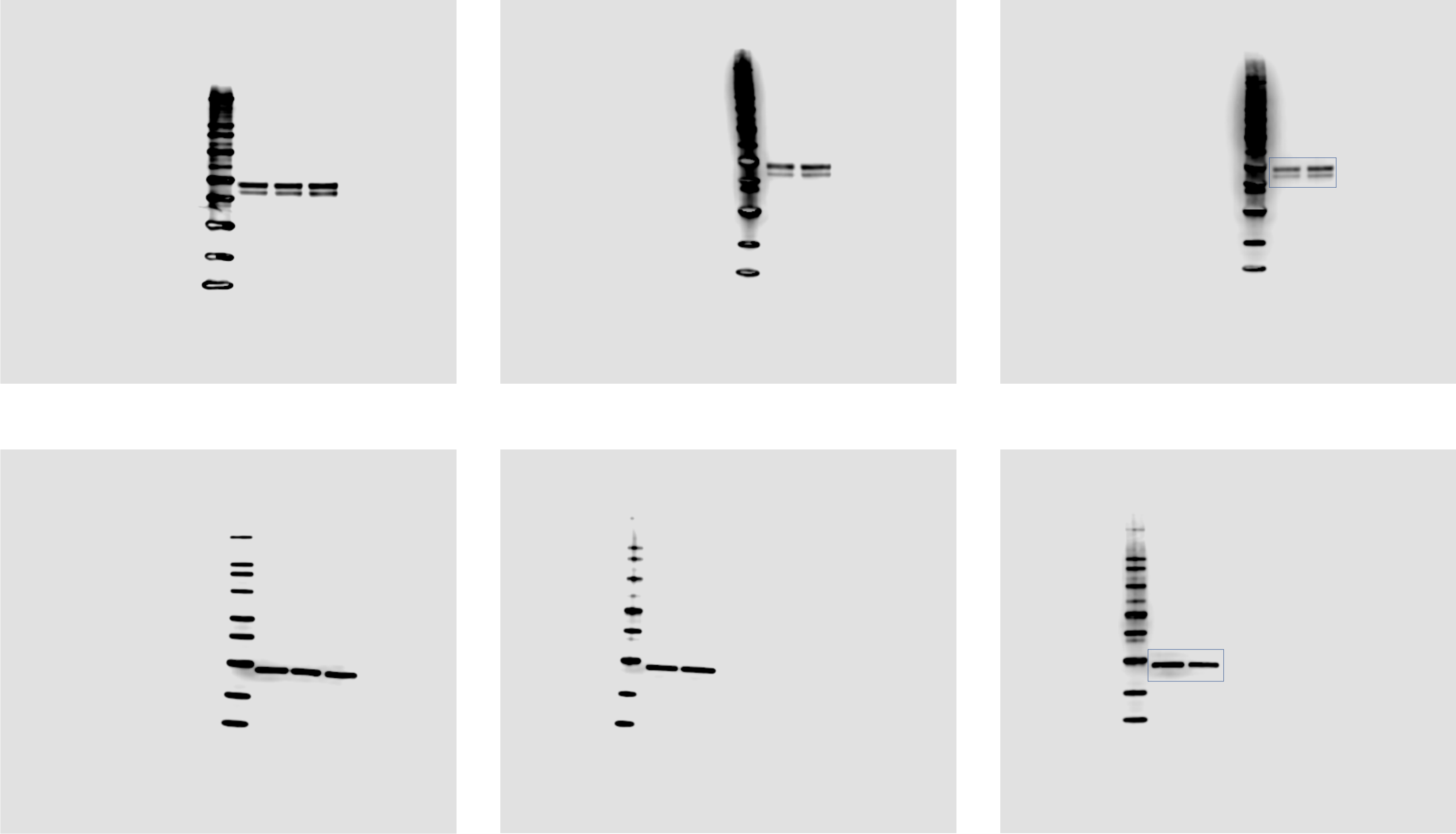

5C, HLE

ERG

GAPDH

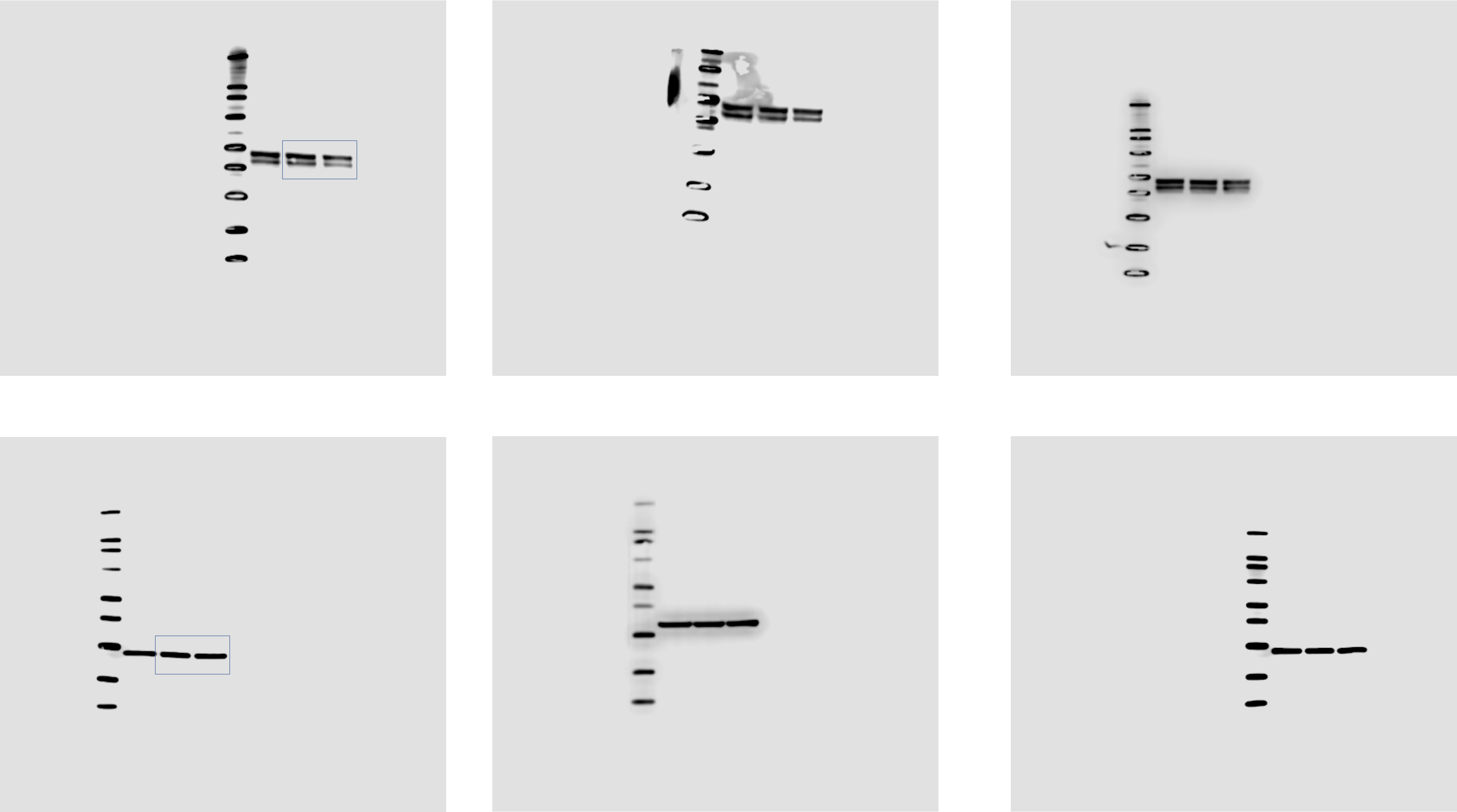

5D, Hep3B

ERG

GAPDH

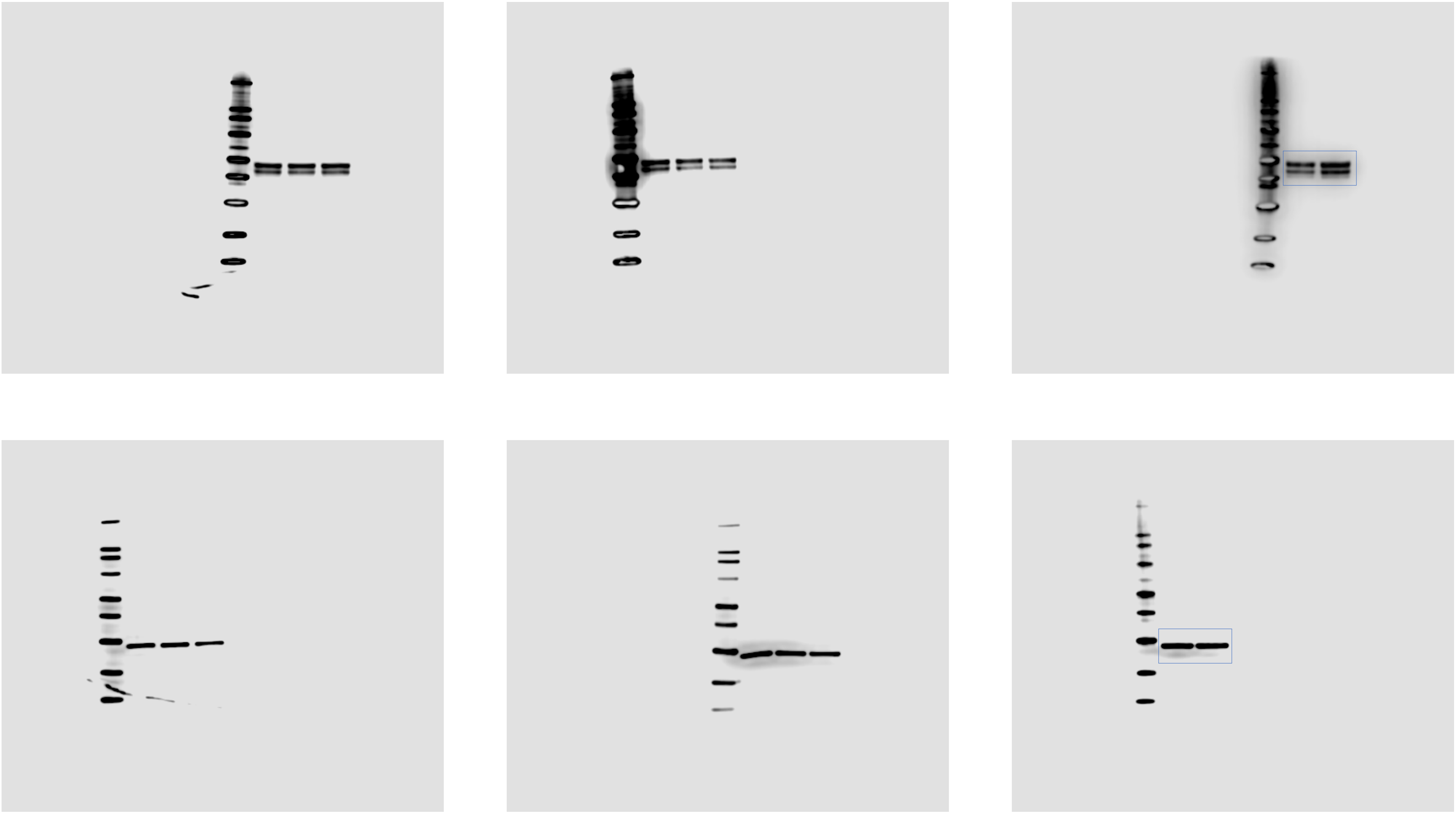

7B, 3HCC Exosomes

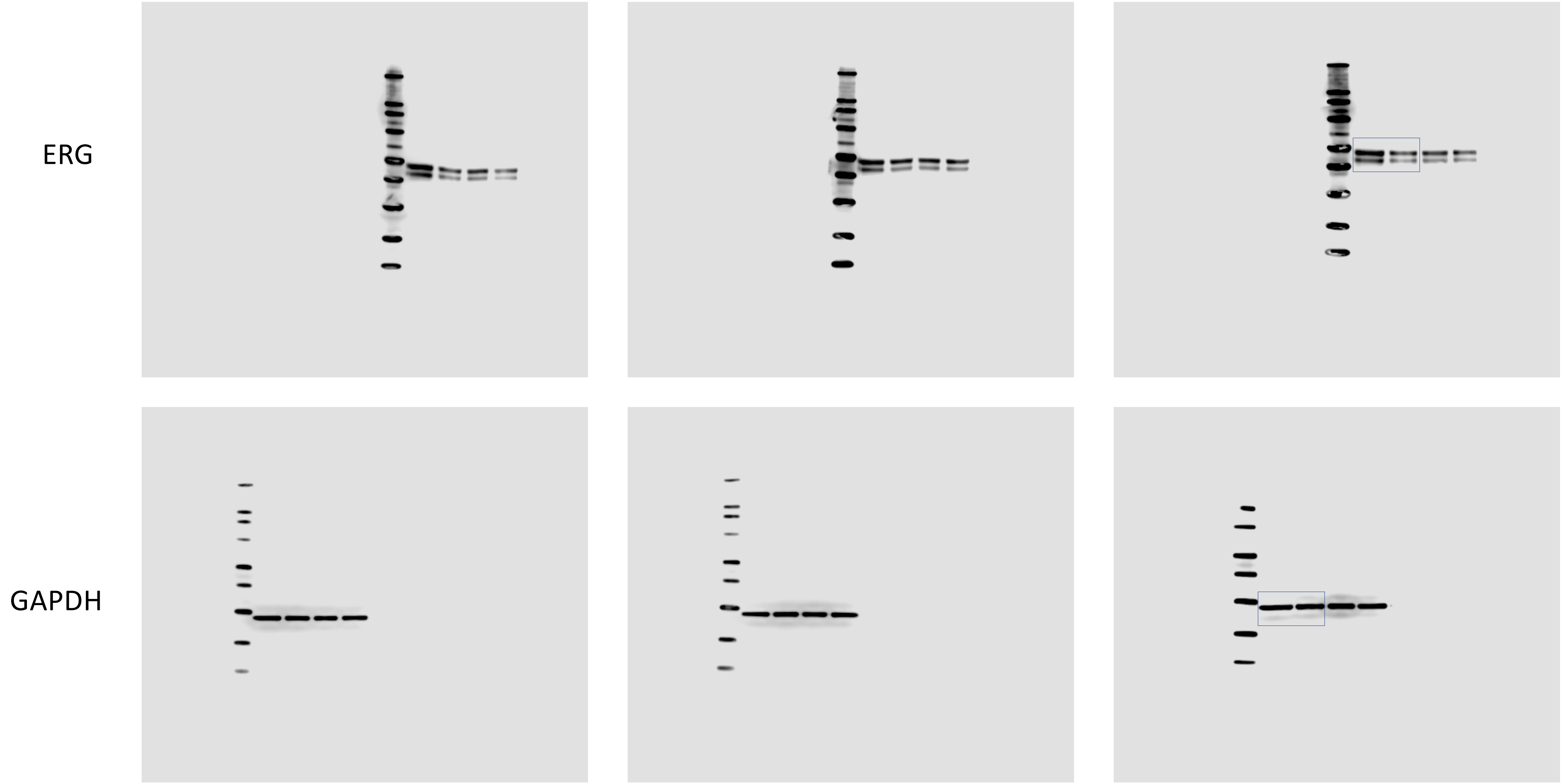

7D, HLE Exosomes

ERG

GAPDH

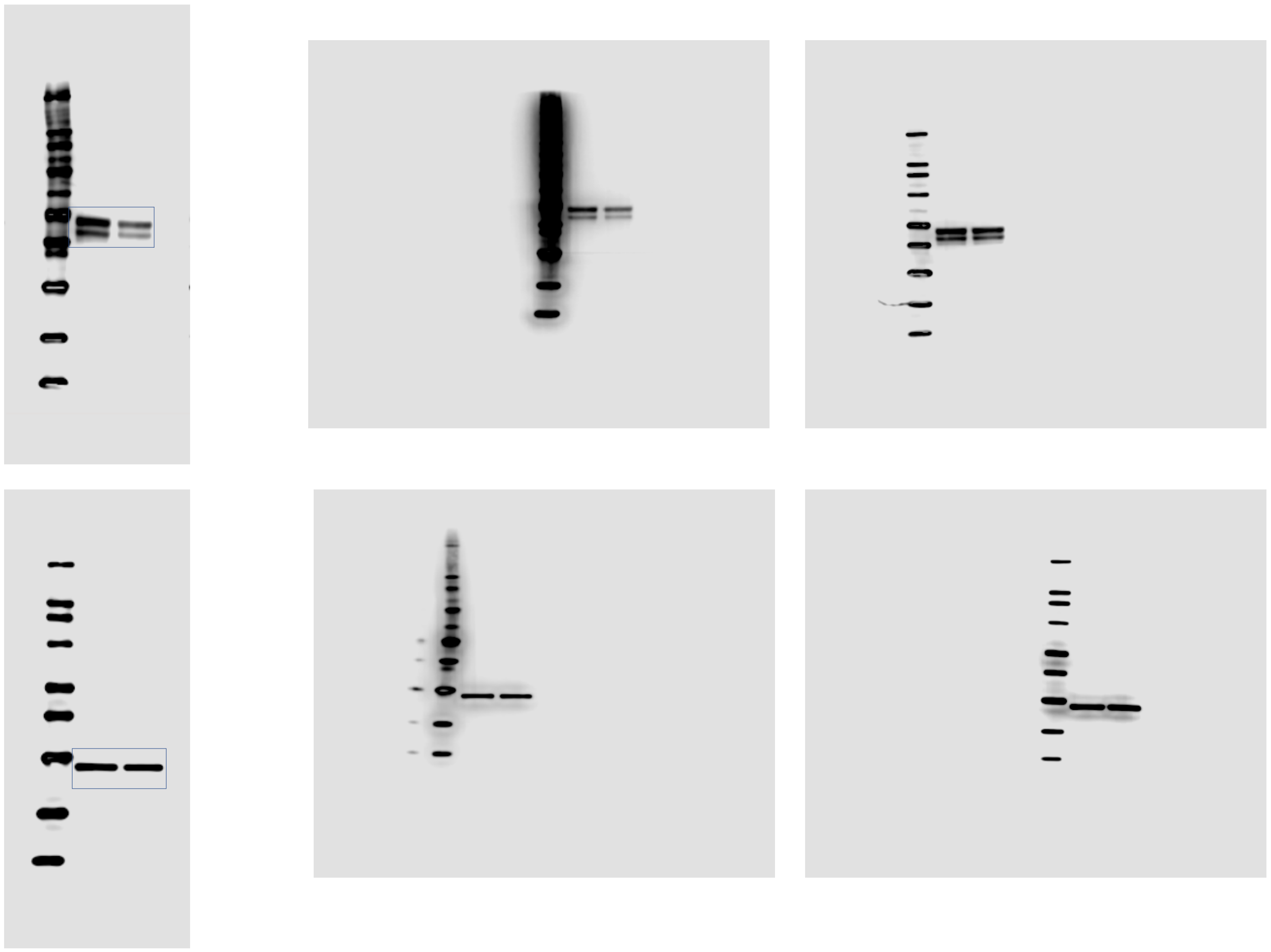

Supplement: Supplementary file 1 — Supplementary information. [file 41598_2020_67425_MOESM1_ESM.pdf]
